# Leptin Regulation of Synaptic Function at Hippocampal TA-CA1 and SC-CA1 Synapses: Implications for Health and Disease

**DOI:** 10.1007/s11064-017-2362-1

**Published:** 2017-08-18

**Authors:** Gemma McGregor, Jenni Harvey

**Affiliations:** Division of Neuroscience, School of Medicine, Ninewells Hospital and Medical School, University of Dundee, Dundee, DD1 9SY UK

**Keywords:** Leptin, Excitatory synaptic transmission, LTP, LTD, Synaptic plasticity, Temporoammonic

## Abstract

Growing evidence indicates that the endocrine hormone leptin regulates hippocampal synaptic function in addition to its established role as a hypothalamic satiety signal. Indeed, numerous studies show that leptin facilitates the cellular events that underlie hippocampal learning and memory including activity-dependent synaptic plasticity and glutamate receptor trafficking, indicating that leptin may be a potential cognitive enhancer. Although there has been extensive investigation into the modulatory role of leptin at hippocampal Schaffer collateral (SC)-CA1 synapses, recent evidence indicates that leptin also potently regulates excitatory synaptic transmission at the anatomically distinct temporoammonic (TA) input to hippocampal CA1 neurons. The cellular mechanisms underlying activity-dependent synaptic plasticity at TA-CA1 synapses differ from those at SC-CA1 synapses and the TA input is implicated in spatial and episodic memory formation. Furthermore, the TA input is an early target for neurodegeneration in Alzheimer’s disease (AD) and aberrant leptin function is linked to AD. Here, we review the evidence that leptin regulates hippocampal synaptic function at both SC- and TA-CA1 synapses and discuss the consequences for neurodegenerative disorders like AD.

## Leptin and the Leptin Receptor

Discovery of the *obese* (*ob*) gene in 1994 via positional cloning techniques enabled insight into the physiological system that controls body weight and energy expenditure [1]. Subsequent investigations identified the *ob* gene product as a 16 kDa protein that reduced food intake and increased energy expenditure in genetically obese (*ob*/*ob*) rodent models, indicating a pivotal role in regulating energy homeostasis. This protein was termed leptin [2, 3].

Leptin is primarily produced and secreted by white adipose tissue and circulates in proportion to adipose mass [3]. The leptin receptor (Ob-R) is encoded by the *diabetes* (*db*) gene [4]. Leptin gains access to the hypothalamus to regulate energy homeostasis via a saturable transport mechanism or by binding to receptors at the blood–brain barrier interface [5]. However recent evidence suggests that leptin can also be made locally within the CNS as leptin mRNA and protein has been detected within the brain [6].

**Fig. 1:**
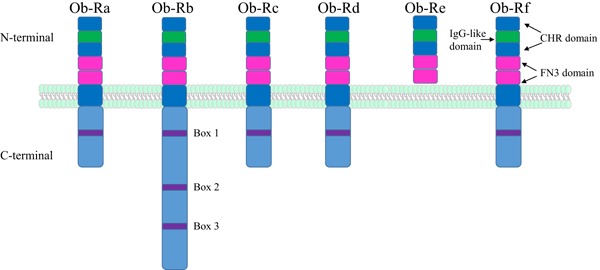
Topology of leptin receptor (Ob-R) isoforms. There are six different isoforms of Ob-R denoted Ob-R_a–f_. Each receptor isoform consists of two cytokine-binding homology regions (CHR1 and CHR2), an IgG-like domain, and two fibronectin type 3 domains (FN3) within its N-terminal. All the isoforms have transmembrane regions, except ObR_e_ which circulates as a soluble leptin binding receptor. The long isoform, Ob-R_b_ is the signalling competent form of the receptor and it contains three intracellular domains (*Box 1–3*) that are required for downstream signalling. Conversely, the short isoforms, Ob_a,c,d,f_ have only one intracellular domain (*Box 1*) and have limited signalling capacity

At least six different isoforms (Ob-R_a–f_) of Ob-R exist (Fig. 1) as a result of alternative splicing of the *db* gene [7, 8]. Each isoform has an identical N-terminal ligand-binding domain but a differential C-terminal region required for signalling. Each isoform gives rise to a single membrane-spanning receptor with the exception of Ob-R_e_ which is thought to circulate as a soluble leptin binding protein. The remaining Ob-R isoforms have either a short intracellular domain containing 30–40 cytoplasmic residues (Ob-R_a,c,d,f_) or a large intracellular domain consisting of 302 residues (known as the long form of the receptor (Ob-R_b_), which is the most signalling competent form of the receptor [9].

## Leptin Receptor Signalling

**Fig. 2:**
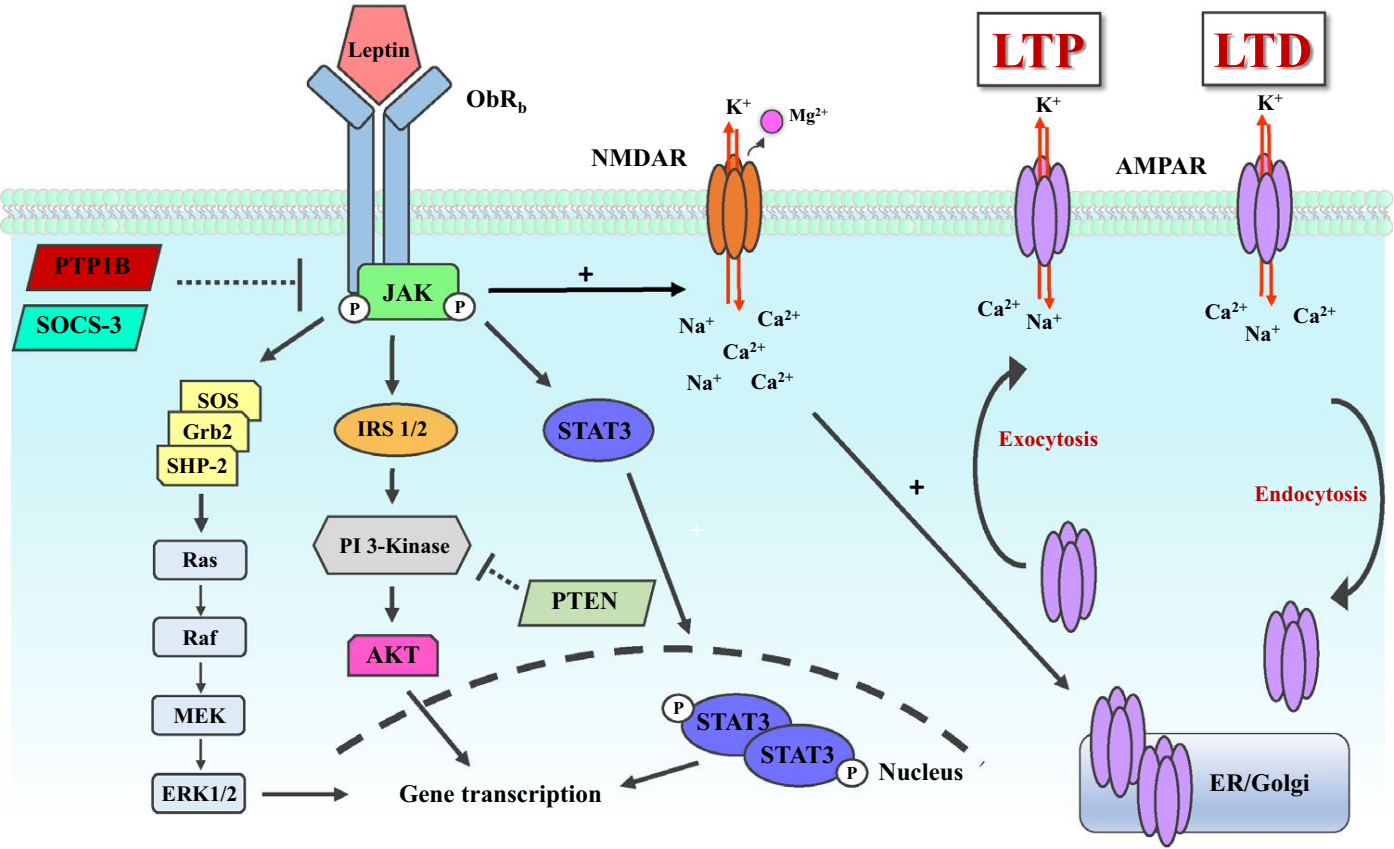
Leptin receptor signalling pathways influence hippocampal excitatory synaptic transmission. Schematic representation of the key Ob-R_b_-driven signalling cascades that are activated following leptin binding to Ob-R_b_. Following leptin binding, phosphorylation of janus activated kinase (JAK2) occurs which leads to dimerization and translocation of signal transducers and activators of transcription (STAT3) to the nucleus. In addition, phosphoinositide 3-kinase (PI 3-kinase)/Akt and ERK signalling cascades are activated following JAK2 phosphorylation culminating in changes in nuclear gene transcription and/or regulation of ion channel function. Ob-R_b_ signalling is inhibited by activation of suppressor of cytokine signalling (SOCS3) and protein-tyrosine phosphatase 1B (PTP1B). Activation of hippocampal Ob-R_b_ facilitates NMDA receptor function, resulting in alterations in AMPA receptor trafficking which in turn promotes persistent changes in hippocampal excitatory synaptic strength

Significant homology exists between Ob-R and the class I cytokine receptor superfamily [4] suggesting possible similarities in the intracellular pathways activated by Ob-R and class I cytokine receptors (Fig. 2). Indeed, like cytokines that signal via interaction with janus tyrosine kinases (JAKs), binding of leptin to Ob-R promotes the recruitment and activation of JAK2 leading to phosphorylation of multiple tyrosine residues (Y985, Y1077 and Y1138) within the cytoplasmic domain of the receptor. JAK2 is constitutively bound to the membrane-proximal part of Ob-R via Box 1 and Box 2 motifs [10]. JAK2 phosphorylation causes transphosphorylation of both JAK2 and Ob-R leading to recruitment of several downstream signalling cascades. Phosphorylation of the tyrosine residue (Y1138) of Ob-R allows binding of the transcription factor, signal transducers and activators of transcription (STAT3) leading to STAT3 dimerization and translocation to the nucleus to regulate gene transcription [11, 12]. In addition to STAT3 activation, insulin receptor substrate (IRS) proteins can be activated as a consequence of cytokine-mediated JAK signalling. One common downstream target of IRS proteins is the p85 subunit of phosphoinositide 3-kinase (PI 3-kinase) [13]. Furthermore, phosphorylation of tyrosine residue (Y985) of Ob-R allows recruitment of tyrosine-protein phosphatase non-receptor type II (SHP-2) which interacts with growth factor receptor-bound protein 2 (Grb2) and Son-of sevenless (Sos) exchange protein. Interaction of Sos with Ras activates a serine signalling cascade whereby Ras–Raf–MEK and mitogen-activated protein kinase (MAPK) are activated, as part of the ERK signalling cascade [14, 15]. In addition cytokine signalling inhibitors, including cytokine inducible sequence (CIS), suppressor of cytokine signalling (SOCS3) and protein tyrosine phosphatase 1B (PTP1B), inhibit leptin signalling by binding to phosphorylated JAK2 [16, 17].

## Extra Hypothalamic Actions of Leptin

In addition to its role in energy homeostasis, leptin is also implicated in the hypothalamic control of bone formation, reproduction and immune function [18–23]. Several lines of evidence indicate a key role for leptin in neuro developmental processes, as significant reductions in brain weight and morphological abnormalities have been observed in leptin deficient (*ob*/*ob*) and insensitive (*db*/*db*) mice: an effect that can be reversed by leptin treatment in *ob*/*ob* mice [24]. In support of a developmental role, a significant surge in leptin occurs during the first two postnatal weeks of development [24], and leptin deficiency delays formation of projections from the arcuate nucleus, thereby implicating leptin in the maturation of hypothalamic circuits during the critical period of development [25].

Although the most well established target for leptin is the hypothalamus, Ob-Rbs are also widely distributed in several extra-hypothalamic regions with high levels of receptor expression detected in the cerebellum and hippocampus [26–28]. In situ hybridisation studies have identified Ob-Rb expression throughout the hippocampal formation [26], as well as in key cortical areas, including the entorhinal cortex, that directly innervates hippocampal CA1 neurons [26, 29]. In addition, hippocampal SC-CA1 synapses express high levels of Ob-Rbs [28] and growing evidence suggests that, in addition to controlling energy expenditure, leptin may regulate hippocampal synaptic function.

## A Role for Leptin in Regulating Hippocampal Excitatory Synaptic Transmission

Our understanding of leptin signalling and its effects on energy homeostasis has been significantly advanced by studying genetically obese rodent models. The identification of spontaneous autosomal recessive mutations within the *db* and *ob* genes has enabled greater insight into the extra-hypothalamic actions of leptin. Zucker *fa*/*fa* rats and *db*/*db* mice have mutations in Ob-R resulting in insensitivity to leptin [30, 31], whereas *ob*/*ob* mice have mutations in the gene that encodes leptin creating a truncated version of the hormone which cannot bind to Ob-R [32]. Rodents possessing these rare mutations develop acute obesity, hyperphagia, heightened metabolic efficiency and develop insulin resistance [31, 32]. Intraperitoneal injection of either mouse or human recombinant leptin can correct the *ob*/*ob* phenotype in rodents, restoring body weight, reducing food intake and increasing energy expenditure [2]. However, neither peripheral nor central administration of leptin reverses the obese phenotype in leptin-insensitive *db*/*db* mice or Zucker *fa*/*fa* rats [2]. Recent studies indicate that genetically obese rodents (*db*/*db* mice, *fa*/*fa* rats) also display impairments in hippocampal-dependent memory processes as marked deficits in spatial memory tasks are observed in the Morris water maze [33, 34]. Furthermore administration of leptin can enhance spatial learning and behavioural performance in wild-type rodents [34, 35]. Moreover, leptin treatment reinstated body weight and neurocognitive performance in a young boy with congenital leptin deficiency [36], suggesting that leptin plays a key role in regulating cognitive function.

It is well established that the strength of communication between excitatory synapses can readily be altered by dynamic changes in the level of neuronal excitation [37]. A persistent increase or decrease in synaptic efficacy is termed long-term potentiation (LTP) or long-term depression (LTD) respectively, and these phenomena are thought to be the key cellular events underlying learning and memory [38–40]. The main excitatory neurotransmitter within the mammalian brain is glutamate which acts on ionotropic α-amino-3-hydroxy-5-methyl-4-isoxazolepropionic acid (AMPA), *N*-methyl-d-aspartate (NMDA) and kainate receptors or metabotropic glutamate receptors (mGluRs) [41].

**Fig. 3:**
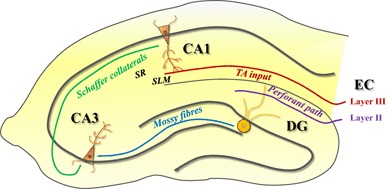
Two anatomically distinct inputs innervate hippocampal CA1 pyramidal neurons. Schematic illustration of neural circuitry of the rodent hippocampus. In the classical tri-synaptic pathway, projections from layer II of the entorhinal cortex (EC) synapse with the dentate gyrus (DG) via the perforant path. Apical dendrites within the stratum radiatum (*SR*) of the CA1 are then innervated by the Schaffer-collateral (SC) fibres which extend from the CA3 region. In contrast, projections from layer III of the EC directly innervate distal dendrites within the stratum lacunosum-moleculare (SLM) and this forms the direct temporoammonic (TA) input to CA1 neurons

Increasing evidence indicates that leptin potently regulates excitatory synaptic transmission at SC-CA1 synapses (Fig. 3). Initial studies found that exposure of acute juvenile (3–5 week old) hippocampal slices to leptin leads to an enhancement in NMDA receptor function and also the conversion of short term potentiation (STP) into LTP [42]. The leptin-driven facilitation of NMDA receptor function requires the activation of PI 3-kinase, ERK and Src tyrosine kinase signalling pathways [42]. In accordance with the potential cognitive enhancing action of leptin, impairments in LTP and LTD have also been detected in hippocampal slices from Zucker *fa*/*fa* rats and *db*/*db* mice [33, 34]. Moreover these synaptic deficits coincide with impaired performance in spatial memory tasks in the leptin-insensitive rodents [33, 35]. Additionally, under conditions of enhanced excitability leptin can induce a novel form of hippocampal LTD in juvenile hippocampal slices. [42]. Leptin-induced LTD requires NMDA receptor, but not mGluR, activation and it occludes low frequency stimulation (LFS)-induced LTD [43] which provides further evidence of a role for leptin in regulating NMDA receptor-dependent synaptic plasticity at SC-CA1 synapses.

In addition, leptin can also reverse (depotentiate) LTP via a process involving activation of NMDA receptors and the calcium/calmodulin-dependent protein phosphatase, calcineurin [44]. It is well known that AMPA receptor trafficking is crucial for activity-dependent synaptic plasticity at hippocampal synapses, such that AMPA receptor insertion into synapses underlies LTP, whereas LTD is associated with AMPA receptor removal from synapses [40, 45]. Increasing evidence indicates that leptin also directly regulates trafficking of AMPA receptors to and from hippocampal synapses [46]. Indeed, leptin-driven depotentiation of hippocampal CA1 synapses involves internalisation of GluA2-lacking AMPA receptors [44]. In contrast, leptin-induced LTP evoked at adult hippocampal SC-CA1 synapses is dependent on the synaptic insertion of GluA2-lacking AMPA receptors [47]. In cultured hippocampal neurons treatment with leptin has distinct effects on different AMPA receptor subunits [48]. At physiological concentrations, leptin preferentially increases the cell surface expression of GluA1, via a process involving NMDA receptor activation. This effect of leptin is associated with phosphorylation and subsequent inhibition of the phosphatase and tensin homolog (PTEN), leading to an increase in intracellular phosphatidylinositol (3,4,5)-trisphosphate (PtdIns(3,4,5)P_3_) levels [47]. Moreover, pharmacological inhibition of PTEN with BpV not only mirrors, but also occludes the effects of leptin on GluA1 trafficking to synapses suggesting a common mechanism of action [47].

Recent studies indicate that the ability of leptin to alter excitatory synaptic transmission at hippocampal SC-CA1 synapses occurs in an age-dependent manner. During the early stages of postnatal development, leptin evokes a transient (P11-18) or persistent (P5-8) synaptic depression that is GluN2B-containing NMDA receptor-dependent and involves activation ERK signalling [49]. This form of LTD induced by leptin at P5-8 occludes LFS-induced LTD, suggesting similar expression mechanisms. Conversely in adulthood, leptin induces a persistent increase in hippocampal synaptic transmission in slices from both adult (12–16 week old) and aged (12–14 month old) animals. Leptin-induced LTP requires activation of GluN2A-containing NMDA receptors and PI 3-kinase signalling and it is also occluded by high frequency stimulation (HFS)-induced LTP [49]. Therefore, not only does leptin play an important role in early postnatal development, but leptin is also a potent regulator of excitatory synaptic function in the adult and ageing hippocampus (Fig. 2).

Previous studies examining the modulatory actions of insulin on synaptic efficacy at SC-CA1 synapses have identified that the ability of insulin to induce either LTP or LTD is highly dependent on the frequency of stimulation [50]. Thus, insulin induces LTD at a stimulation frequency of 0.033 Hz, whereas LTP was induced by insulin when the frequency of stimulation was increased to 10 Hz [50], suggesting that insulin alters the frequency response curve of activity-dependent synaptic plasticity. In contrast, the bi-directional effects of leptin on excitatory synaptic strength appear to be independent of stimulation frequency as the ability of leptin to induce LTD at P5-8 or LTP at adult SC-CA1 synapses occurs during low frequency simulation (0.033 Hz).

### Leptin Regulation of Neuronal Morphology

Marked changes in the structure and density of hippocampal dendrites and spines accompany activity-dependent synaptic plasticity and these alterations are thought to play a role in maintaining the resulting changes in synaptic efficacy. Several hormones can induce rapid structural changes in neuronal morphology which provides an additional route for regulating neuronal connectivity and excitatory synaptic strength. Leptin is also implicated in regulating neuronal morphology as reductions in hippocampal spine density are evident in leptin-insensitive (*db*/*db*) mice compared to wild type littermates [51]. The ability of leptin to promote spine formation involves activation of the CaM kinase signalling pathway and subsequent trafficking of TrpC channels to the plasma membrane [52]. Moreover, in cellular studies exposure of hippocampal neurons to leptin results in rapid and significant alterations in the number and motility of dendritic filopodia, which mirrors the initial stages in spine formation [53]. Blockade of NMDA receptors with D-AP5 or synaptic activity with TTX prevents the effects of leptin, suggesting that synaptic activation of NMDA receptors underlies the leptin-driven changes in hippocampal dendritic morphology [53]. Alterations in hippocampal neuron morphology have also been reported in vivo following dietary changes in leptin levels. Indeed, mice subjected to a high fat diet displayed not only elevated leptin levels, but also an increase in dendritic spine density in hippocampal CA1 neurons [54].

## The Temporoammonic (TA)-CA1 Synapse

Although extensive evidence indicates that leptin regulates hippocampal synaptic function, most studies have focused on its modulatory actions at SC-CA1 synapses. However, it is known that pyramidal neurons within the hippocampal CA1 region receive two distinct inputs from the entorhinal cortex (EC; Fig. 3). The classical tri-synaptic pathway originates in layer II of the EC and projects to the dentate gyrus (DG) and the CA3 region via the perforant path. The stratum radiatum contains the SC pathway which originates in the CA3 region and is the indirect input into the CA1 region. However, the temporoammonic (TA) input originates from layer III of the EC and directly innervates the stratum lacunosum-moleculare of the CA1 region [55, 56]. These two inputs to CA1 neurons not only differ in receptor and ion channel composition but also in the mechanisms underlying activity-dependent synaptic plasticity evoked at these synapses [56]. Indeed, the stratum lacunosum-moleculare where TA-CA1 synapses terminate, express higher levels of dopamine receptors and a larger glutamatergic NMDA component than the SC input [57, 58]. Furthermore, monoamines such as dopamine, strongly depress excitatory synaptic transmission at TA-CA1 synapses, with little effect at SC-CA1 synapses [57, 59, 60]. In addition, distinct presynaptic release mechanisms have been reported for TA-CA1 compared to SC-CA1 synapses [61]. Indeed, functional imaging of presynaptic release kinetics has identified that TA-CA1 synapses have a lower efficacy of vesicle release than SC-CA1 synapses, and consistent with this, TA-CA1 synapses also display a much larger paired pulse ratio than SC-CA1 synapses [61]. Differences in the contribution of N-type voltage gated Ca^2+^ channels to vesicle release mechanisms has been identified as a key factor underlying the differing efficacies of release at the two inputs onto CA1 neurons [61].

## Long Term Potentiation (LTP) at TA-CA1 Synapses

In addition to the reported differences in synaptic plasticity at TA- and SC-CA1 synapses, the TA input also regulates activity-dependent synaptic plasticity at SC-CA1 synapses. Indeed, time-dependent bursts of TA activity modulates the probability of SC-CA1 evoked spikes and significantly reduces the magnitude of potentiation at SC-CA1 synapses [62]. NMDA receptor dependent-LTP is readily evoked at TA-CA1 synapses using a high-frequency stimulation paradigm (100 Hz, 1 s), and TA-CA1 LTP occurs independently of changes to SC-CA1 plasticity but requires severance of the hippocampal CA3 region to isolate the TA input [60, 62]. Studies using hippocampal slices obtained from 6 to 7 week old animals demonstrate that HFS induces both early- and late-phase LTP which requires activation of voltage-gated Ca^2+^ channels (VGCC) and NMDA receptors [63]. The same study found that TA-CA1 LTP was insensitive to GABA_A_ receptor blockade but was dependent on GABA_B_ receptor activation [63]. In slices from adult mice, VGCCs and NMDA receptors are also implicated in the induction of activity-dependent LTP at TA-CA1 synapses [61]. However, in contrast to SC-CA1 synapses, LTP at TA-CA1 synapses involves a presynaptic mechanism that depends on an increase in release efficacy due to recruitment of N-type VGCCs [61]. Conversely, in juvenile (P11-18) hippocampal slices, HFS-induced LTP at TA-CA1 synapses requires activation of postsynaptic NMDA receptors and is dependent on ERK, but not PI 3-kinase, signalling [60]. Furthermore, GluA2-lacking AMPA receptors are required for the maintenance, but not induction of HFS-induced LTP at juvenile TA-CA1 synapses [59]. Recent in vivo studies demonstrate that LTP that lasts in excess of 24 h can be evoked by tetanic stimulation in freely behaving rats [56, 64]. Further investigation found that this form of *in vivo* LTP is dependent on NMDA receptor activation [64]. Moreover, in hippocampal slices (P30-50), stimulation of proximal TA inputs induces LTP at distal SC-CA1 synapses when the two inputs are paired at a precise time interval [65]. This form of heterosynaptic plasticity requires activation of NMDA receptors and inositol triphosphate (IP_3_) receptor-dependent release of intracellular Ca^2+^ [65]. In addition, distinct differences in short-term facilitation have been observed at TA-CA1 synapses and SC-CA1 synapses, which provides further evidence that there are key differences in presynaptic function at the two synaptic inputs to CA1 neurons [66]. Thus, although both the TA and SC inputs have distinct synaptic plasticity mechanisms, the anatomically distinct inputs are likely to act in concert to regulate hippocampal synaptic function. However, as activity-dependent synaptic plasticity can be readily and independently induced at TA-CA1 synapses this suggests that this pathway also plays a fundamental role in hippocampal information processing.

## Long Term Depression at TA-CA1 Synapses

In addition to LTP, activity-dependent LTD has also been observed at the TA input to CA1 neurons [67]. Thus, in slices from 6 to 7 week old animals, LFS (1 Hz, 10 min) readily induces robust LTD which lasts over 1 h. Induction of TA-CA1 LTD requires NMDA receptor activation but is unaffected following blockade of either GABA_A_ or GABA_B_ receptors [67]. In contrast, a distinct form of activity-dependent LTD has been reported in slices from 3 to 4 month old rats that is dependent on GABA_B_ and kainate receptor activation [68]. Furthermore, recent studies indicate that LFS (1 Hz, 15 min) induces NMDA receptor-dependent LTD at TA-CA1 synapses in slices from 3 to 6 month old adult animals [69]. TA-CA1 LTD is independent of PI 3-kinase or ERK signalling but requires activation of the canonical JAK2–STAT3 signalling cascade and rapid gene transcription [69]. Furthermore in parallel studies, chemical (NMDA; 20 μM; 10 min) induction of LTD in cultured hippocampal neurons reduces the cell surface expression of GluA1-containing AMPA receptors; an effect that is accompanied by a simultaneous increase in the phosphorylation of JAK2 and STAT3 [69], indicating involvement of JAK–STAT signalling in AMPA receptor internalisation and LTD. JAK–STAT signalling is also implicated in NMDA receptor-dependent LTD at juvenile SC-CA1 synapses, however in contrast to TA-CA1 synapses, LTD evoked at SC-CA1 synapses does not require gene transcriptional changes [70].

## Leptin Regulates Excitatory TA-CA1 Synapses

Increasing evidence indicates that the TA input plays a fundamental role not only in activity-dependent synaptic plasticity at CA1 synapses, but also hippocampal dependent memory processes [62, 71–74]. Recent studies indicate that leptin also modulates excitatory synaptic transmission at TA-CA1 synapses [60], however there are clear differences in the regulatory actions of leptin at TA-CA1 and SC-CA1 synapses. Indeed, application of leptin induces a novel form of LTP at juvenile TA-CA1 synapses [60], which contrasts with the synaptic depression induced by leptin at SC-CA1 synapses at the same age [49]. Leptin-induced LTP at juvenile TA-CA1 synapses is NMDA receptor-dependent and requires selective activation of GluN2B subunits. Furthermore, activation of PI 3kinase, but not ERK, signalling and subsequent insertion of GluA2-lacking AMPA receptors is required for leptin-induced LTP at TA-CA1 synapses [60]. Moreover, HFS-induced LTP occludes leptin-evoked LTP at the TA-CA1 synapses and vice versa suggesting common expression mechanisms underlie both forms of synaptic plasticity [60].

Collectively, these data indicate not only that leptin has opposing effects on excitatory synaptic transmission at SC-CA1 and TA-CA1 synapses, but also that distinct cellular mechanisms underlie the ability of leptin to modulate synaptic efficacy at these anatomically distinct synaptic connections [49, 60]. Previous studies have demonstrated that at SC-CA1 synapses, the polarity of synaptic modulation by leptin varies significantly with age and is also highly dependent on the molecular composition of NMDA receptors [49]. Indeed, the role of different NMDA receptor subunits in mediating the age-dependent effects of leptin parallels the developmental switch that occurs in the expression of NMDA receptor subunits from predominantly GluN2B-containing to GluN2A-containing subunits in the hippocampus [75, 76]. This has led to the suggestion that activation of molecularly distinct NMDA receptor subunits that couple to specific signalling pathways is required for leptin-driven alterations in excitatory synaptic strength at SC-CA1 synapses [49]. However, although leptin-induced LTD at SC-CA1 synapses and leptin-induced LTP at TA-CA1 synapses both involve GluN2B activation, divergent signalling cascades are implicated in these leptin-driven events [49, 60]. As there are clear differences in the expression of ion channels and receptors, including NMDA receptors at TA-CA1 and SC-CA1 synapses [61, 63, 65, 66, 77], it is likely that the localisation and/or molecular composition of NMDA receptors is a key factor in determining not only the signalling pathways that are activated, but also the polarity of leptin’s effects on synaptic function.

Although the effects of leptin on excitatory synaptic transmission at adult TA-CA1 synapses has not yet been examined, in view of the reported bi-directional actions of leptin in juvenile hippocampus [49], it is feasible that opposing actions of leptin will also occur at the anatomically distinct CA1 synapses in adult hippocampus (see Table 1). Thus, in contrast to LTP induced by leptin at adult SC-CA1 synapses, it is possible that application of leptin results in the induction of LTD at TA-CA1 synapses in adulthood. Moreover, as the sensitivity of SC-CA1 synapses to leptin declines with age [49], a similar reduction in leptin sensitivity may also occur at TA-CA1 synapses, such that the magnitude of LTD evoked by leptin at aged TA-CA1 synapses is markedly attenuated. As PI 3-kinase activation underlies leptin-induced LTP at both juvenile TA-CA1 and adult SC-CA1 synapses, respectively (see Table 1), it is also possible that analogous signalling cascades underlie leptin-induced LTD at both synapses. Thus, as ERK signalling is implicated in leptin-induced LTD at juvenile SC-CA1 synapses, ERK may also play a prominent role in leptin-induced LTD at adult TA-CA1 synapses.

**Table 1:** Summary of the age-dependent and bi-directional effects of leptin on synaptic efficacy at TA- and SC-CA1 synapses

*TA-CA1 synapse*	*SC-CA1 synapse*
Juvenile (P11-18) hippocampus	
Leptin-induced LTP	Leptin-induced LTD
GluN2B-dependent	GluN2B-dependent
PI 3-kinase signalling	ERK signalling
Insertion of GluA2-lacking AMPA receptors	Removal of GluA2 lacking AMPA receptors
Adult (3–6 month) hippocampus	
Leptin-induced LTD	Leptin-induced LTP
GluN2A dependent?	GluN2A dependent
ERK signalling?	PI-3K signalling
Removal of GluA2 lacking AMPA receptors	Insertion of GluA2-lacking AMPA receptors

Summary table highlighting the divergent actions of leptin at hippocampal TA-CA1 and SC-CA1 synapses. In juvenile hippocampus, a novel form of LTD is induced by leptin that requires GluN2B activation and involves ERK-dependent signalling. Conversely, at juvenile TA-CA1 synapses, leptin induces LTP that involves activation of GluN2B-containing NMDA receptors and PI 3-kinase. In contrast to its actions in juvenile tissue, leptin induces LTP at adult SC-CA1 synapses; a process that is GluN2A-dependent and involves PI 3-kinase driven insertion of GluA2-lacking AMPA receptors. Although not yet determined, it is speculated that leptin will induce a novel form of LTD at adult TA-CA1 synapses, that is likely to be GluN2A dependent and may involve ERK-driven removal of GluA2 from synapses

### A Link Between Leptin and Alzheimer’s Disease (AD)

It is well-established that neurodegenerative diseases like Alzheimer’s disease (AD) result in impairments in cognitive function, information processing and subsequent memory loss. Although there are some genetic risk factors for AD, most cases are sporadic with the underlying etiology of the disease unknown [78]. However evidence suggests that diet and lifestyle markedly influence the risk of developing AD [79]. Recent studies support a link between leptin levels and AD. Indeed, clinical evidence has identified that the risk of AD is significantly increased with mid-life obesity. Resistance to leptin and/or dysfunctions in the leptin system may contribute to this risk as it is well established that obesity is due to development of leptin resistance. Clinical studies have found good correlation between weight loss and AD progression and eventual mortality [80]. Moreover significant reductions in the serum leptin levels have been detected in AD patients [81, 82]. A prospective study by Leib et al. [83] also found a link between leptin and the incidence of AD as individuals that had high plasma levels of leptin but were not obese, had a significantly lower incidence of AD than those with low leptin levels.

Although most rodent models of AD do not fully replicate the pathological and behavioural characteristics of human AD, attenuated circulating leptin levels have also been detected in various AD models [84, 85]. Moreover, a transgenic AD mouse model (APP/PS1) with elevated toxic amyloid-β (Aβ) plaques and memory loss, displays a reduction in Ob-R levels as well as key components of Ob-R signalling, including STAT3 and SOCS3 [86].

It is well known that age is a key risk factor for AD, and that dysfunctions in metabolic systems occur during normal ageing. In accordance with this, age-related alterations in the leptin system have been widely reported. Indeed, aged wild-type rats exhibit a reduction in Ob-R expression and an increase in SOCS3 and PTP1B [86–92]. The sensitivity of hippocampal SC-CA1 synapses to leptin is also markedly reduced with age [49]. This suggests that impairments in the leptin system may occur prior to Aβ plaque formation. Indeed, application of leptin restricts Aβ production and reduces the toxic burden of Aβ in AD-transgenic rodents models [91]. Consequently, impairment or age-related changes in the leptin system are likely to limit these protective actions of leptin and boost Aβ production. In addition, alterations in key Ob-R-related signalling pathways have been identified in various AD models. Thus, the levels of phosphorylated STAT3 are age-dependently reduced in a rodent model of AD whereas inhibition of JAK2–STAT3 signalling by Aβ induces a significant loss in spatial working memory [93, 94]. Together this suggests that dysfunctions in the leptin system are associated with AD, and that boosting the central actions of leptin may have therapeutic benefit.

In support of the therapeutic potential of leptin, increasing evidence indicates that exposure to leptin prevents both the acute and chronic actions of Aβ. A number of studies have shown that acute exposure of hippocampal slices to Aβ results in impairments in activity-dependent hippocampal synaptic plasticity, such that Aβ blocks the induction of LTP, but enhances LTD [95, 96]. In addition, Aβ interferes with glutamate receptor trafficking processes such that internalisation of GluA1 and GluA2 occurs after treatment of hippocampal neurons with Aβ [97–99]. Recent studies indicate that leptin prevents the detrimental effects of Aβ on hippocampal synaptic plasticity and glutamate receptor trafficking. Thus, application of leptin rescues Aβ-induced inhibition of LTP and facilitation of LTD [98, 99]. Furthermore, treatment with leptin prevents Aβ-driven internalisation of the AMPA receptor subunit, GluA1 [98, 99]. In addition to preventing the acute effects of Aβ, leptin also protects against the chronic actions of Aβ that result in neuronal cell death. In cortical neurons, leptin reduces neuronal cell death induced by either Aβ or Cu^2+^ ions, and it attenuates Aβ-driven upregulation of endophilin 1 and phosphorylated tau [98]. The levels of endophilin 1 and phosphorylated tau are also regulated by leptin as the levels of both proteins are significantly enhanced in cortical tissue from leptin-insensitive Zucker *fa*/*fa* rats [98]. Moreover, crossing leptin deficient or insensitive mice (*ob*/*ob* or *db*/*db*) with AD transgenic mouse models (APP23 or *tau*^*P301L*^) exacerbates AD-related pathology thereby providing further evidence that impairments in the leptin system may accelerate AD neurodegeneration [100, 101].

It is well established that the EC and hippocampus are two of the most severely affected brain regions, with intraneuronal changes occurring in the earliest stage of AD [102]. Histological studies indicate that the TA input undergoes significant morphological changes as a consequence of ageing but also in AD [103]. Indeed, reductions in myelin staining and loss of synapses in this region have been observed in two pre-clinical models of tauopathy (tau^P301L^ and tau^P301L^ co-expressing GSK3β) [104]. Moreover, in an AD mouse model overexpressing mutant human tau, deficits in synaptic plasticity at TA-CA1 synapses have been detected [105]. TA-CA1 synapses are thought to play a key role in spatial novelty detection, intermediate-term working memory as well as memory consolidation and remote memory retrieval [71, 72, 74, 106]. The TA input to CA1 neurons is also implicated in integrating cortical and place cell information, thereby contributing to the formation of episodic memories [73]. Therefore, as TA-CA1 synapses are a target for the early pathological changes as well as the synaptic impairments that occur in AD, it is feasible that degeneration within the TA pathway contributes to episodic memory deficits occurring in the early stages of AD. Thus, as leptin levels are linked to AD risk, it is vital that there is greater understanding of how the ability of leptin to regulate TA-CA1 synapses alters with age and also in AD models.

## Conclusions

In conclusion, it is now well established that the endocrine hormone leptin plays a pivotal role in regulating excitatory synaptic transmission at SC-CA1 synapses. However recent studies indicate that the anatomically distinct TA input to CA1 synapses, which is an early site for degeneration in AD, is also tightly regulated by leptin. As accumulating evidence links leptin levels to the risk of AD, and leptin has reported therapeutic benefit in AD models, the ability of leptin to regulate TA-CA1 synapses has important implications for the role of leptin in health and neurodegenerative disorders like AD.
